# Left Atrial Strain Imaging by Speckle Tracking Echocardiography: The Supportive Diagnostic Value in Cardiac Amyloidosis and Hypertrophic Cardiomyopathy

**DOI:** 10.3390/jcdd10060261

**Published:** 2023-06-15

**Authors:** Ines Paola Monte, Denise Cristiana Faro, Giancarlo Trimarchi, Fabrizio de Gaetano, Mariapaola Campisi, Valentina Losi, Lucio Teresi, Gianluca Di Bella, Corrado Tamburino, Cesare de Gregorio

**Affiliations:** 1 Department of Surgery and Medical-Surgical Specialties, University of Catania, Via Santa Sofia 78, 95123 Catania, Italy; denisefaro88@gmail.com (D.C.F.); fabridegafp7@gmail.com (F.d.G.); vale.losi@gmail.com (V.L.); tambucor@unict.it (C.T.); 2 Department of Clinical and Experimental Medicine, University Hospital of Messina, 98121 Messina, Italy; giancarlo.trimarchi18@gmail.com (G.T.); lucioteresi@gmail.com (L.T.); gianluca.dibella@unime.it (G.D.B.); cdegregorio@unime.it (C.d.G.); 3 Azienda Ospedaliera Provinciale di Catania, Santa Maria e Santa Venera Hospital, 95024 Acireale, Italy; campisi.mariapaola@gmail.com

**Keywords:** Cardiac Amyloidosis, left-ventricle hypertrophy, diastolic dysfunction, heart failure, hypertrophic cardiomyopathy, speckle tracking echocardiography, atrial strain, amyloid atrial myopathy

## Abstract

**Background**: Left atrial (LA) function is crucial for assessing left ventricular filling in various cardiovascular conditions. Cardiac Amyloidosis (CA) is characterized by atrial myopathy and LA function impairment, with diastolic dysfunction up to restrictive filling pattern, leading to progressive heart failure and arrhythmias. This study evaluates LA function and deformation using speckle tracking echocardiography (STE) in patients with CA compared to a cohort of patients with sarcomeric Hypertrophic Cardiomyopathy (HCM) and a control group. **Methods**: We conducted a retrospective, observational study (from January 2019 to December 2022) including a total of 100 patients: 33 with ATTR-CA, 34 with HCMs, and 33 controls. Clinical evaluation, electrocardiograms, and transthoracic echocardiography were performed. Echocardiogram images were analyzed in post-processing using EchoPac software for LA strain quantification, including LA-reservoir, LA-conduit, and LA-contraction strain. **Results**: The CA group exhibited significantly impaired LA function compared to HCMs and control groups, with LA-reservoir median values of −9%, LA-conduit −6.7%, and LA-contraction −3%; this impairment was consistent even in the CA subgroup with preserved ejection fraction. LA strain parameters correlated with LV mass index, LA volume index, E/e’, and LV-global longitudinal strain and were found to be associated with atrial fibrillation and exertional dyspnea. **Conclusions**: LA function assessed by STE is significantly impaired in CA patients compared to HCMs patients and healthy controls. These findings highlight the potential supportive role of STE in the early detection and management of the disease.

## 1. Introduction

The assessment of left atrial (LA) morphology and function is paramount in gauging left ventricular filling amidst a variety of cardiovascular conditions, encompassing hypertension, heart failure, valvular heart disease, and cardiomyopathies.

The LA carries out three discrete functional phases, each accounting for approximately 50%, 30%, and 20% of left ventricle (LV) filling in healthy subjects, respectively.

The “reservoir” phase of the LA transpires during the isovolumetric contraction, ejection, and relaxation phase of the LV, with the mitral valve in a closed state. During this phase, the LA serves as a storage unit for potential energy.

The “conduit” phase occurs in the initial phase of ventricular diastole, beginning with the opening of the atrioventricular valves and continuing until the commencement of LA contraction. Throughout this phase, the LA functions as a pathway for blood from the pulmonary veins to the LV.

Lastly, during the “contraction” or “pump” phase, the contraction of the LA aids in augmenting left ventricular filling [1].

LA function is vital in averting heart failure as the LA initiates its inherent compensatory mechanisms in the event of left ventricular dysfunction. LA dilatation, as signified by a volume index (LAVi) exceeding 34 mL/m2, is a crucial indicator of LV filling and diastolic function [2].

Speckle tracking echocardiography (STE) can be used to investigate LA function, and LA strain, as quantified via the LA strain curve with two positive peaks corresponding to LA-reservoir and LA-pump function, serves as a sensitive indicator of LV filling pressure, superior to LA-volume and utilized for the early detection of preclinical LV dysfunction and remodeling [3,4]. Recent research suggests that atrial fibrosis precipitates impaired atrial contractility, preceding atrial remodeling, which forecasts cardiovascular morbidity and mortality [5].

LA dysfunction is influenced by LV remodeling and diastolic dysfunction amidst various cardiovascular disorders, including cardiomyopathies. A defining characteristic of restrictive cardiomyopathies is myocardial rigidity, denoted by diastolic dysfunction, hindering LV filling and conserving LV systolic function and LA enlargement/remodeling. Patients diagnosed with Sarcomeric Hypertrophic Cardiomyopathy (HCMs) are especially susceptible to adverse LA remodeling owing to heterogeneous myocardial hypertrophy, LV systolic and diastolic dysfunction, impaired LV global longitudinal strain (LV-GLS) and heightened mechanical dispersion [6], and progressive LA enlargement accompanied by morpho-functional impairment. This arises as a result of increased LV filling pressure, mitral insufficiency, outflow tract obstruction, and progressive LA fibrosis, culminating in what is referred to as “atrial myopathy”. Certain studies have demonstrated that LA strain values in HCM are inferior to those in healthy subjects and are associated with adverse outcomes [7].

Cardiac Amyloidosis (CA) is induced by the intramyocardial deposition of abnormally folded amyloid fibrils, typically monoclonal immunoglobulin light chains in the context of systemic amyloidosis (AL) or transthyretin (ATTR), either in its hereditary (ATTRv) or acquired wild type (ATTRwt) form. This results in a steady increase in LV thickness, diastolic dysfunction, elevated filling pressures, and progressive LA dilatation and dysfunction [8,9,10]. Escalating LA-size has been correlated with adverse outcomes in patients with CA [11]. LA strain parameters, incorporating reservoir, conduit, and booster pump function, were compromised in individuals with CA and correlated well with the degree of LV dysfunction [12].

The aim of this study is to evaluate LA function and deformation using strain by STE in patients with CA compared to a cohort of patients with sarcomeric HCM and a control group and correlate the three LA strain parameters with echo (LV-mass, diastolic and systolic function, and LV-GLS) and clinical parameters. In addition to previous data from literature, we conducted this study to deepen the current knowledge by analyzing a subgroup of CA patients with preserved ejection fraction (pEF) to mitigate any potential influence on the echocardiographic and clinical parameters of diastolic dysfunction caused by reduced contractile function (systolic dysfunction), which is characteristic of advanced staged of Cardiac Amyloidosis. The goal was to make a comparison with HCMs, using a more similarly selected population in terms of EF.

## 2. Materials and Methods

### 2.1. Study Population

Our retrospective, observational study included patients aged over 18 years, who were referred to our Cardiology Units from January 2019 to December 2022. Patients were selected based on the following inclusion criteria and divided into two groups:

1. Patients affected by Transthyretin Cardiac Amyloidosis (ATTR), caused either by genetic mutation or wild type, diagnosed in accordance with the 2021 European Society of Cardiology (ESC) position statement [8]—using imaging criteria (transthoracic echocardiography [TTE] or cardiac magnetic resonance [CMR]) and total body 99mTc-PYP, DPD, or HMDP bone scintigraphy with SPECT (Perugini 2 or 3), after ruling out light chains amyloidosis (AL).
2. Patients diagnosed with Sarcomeric Hypertrophic Cardiomyopathies (HCMs), in line with the ESC 2014 guidelines [13], featuring TTE criteria: maximum wall thickness ≥15 mm (or ≥13 mm for family members), irrespective of the identification of the genetic mutation. Patients presenting with Obstructive HCM and LVOT gradient >30 mmHg were excluded from the analysis.

A control group (Co) of similar age range was introduced for comparison. The controls did not report any cardiac symptoms in their daily life, nor did they have pathological alterations found during physical examination, electrocardiogram (ECG), or basal echo or any organ damage. Some of them, consistent with the age range they belong to, were found during the visit to have office-measured borderline blood pressure values (normal-high) or to be within the range of grade 1 hypertension.

Upon enrolment, a thorough clinical evaluation was conducted, inclusive of a cardio-myopathy-oriented medical history and a comprehensive assessment of cardiovascular risk factors and major comorbidities. We executed ECG, 2D-color Doppler TTE, and dynamic-ECG (or ICD interrogation).

From the original database, we included all patients with optimal image quality (owing to an adequate acoustic window and/or patient’s cooperation), suitable for speckle tracking analysis.

### 2.2. Echocardiography

Echocardiography was performed using an E95-GE machine equipped with a 1.5- and 3.6-MHz transducer, with a thorough assessment of parameters (chamber dimensions, systolic and diastolic function, and global longitudinal strain by STE) conforming to current recommendations [2,3,14]. Post-processing image analysis was performed using semi-automatic software (EchoPAC, ver. 2.02, GE, Chino, CA, USA) to achieve LV and LA strain quantification. GLS was analyzed from the apical views, at 60–70 fpm, from the average of 3 consecutive cardiac cycles.

### 2.3. LA Strain Assessment

The evaluation of LA strain was performed following the most recent EACVI consensus papers [3,15]. They advocate that it should be executed through dedicated image acquisition, from an LA-focused view (4 chambers or 2 chambers), with a narrow image sector, ensuring non-foreshortened images of the LA and acquiring 3–5 consecutive, regular beats. A high-quality ECG trace with a visible *p* wave is essential, and the acquisition of mitral and aortic valve Doppler waves can provide better retrospective definition of time intervals. Dedicated software should be utilized whenever possible. The region of interest (ROI) is defined by delineating the endocardial contour and encompassing the LA myocardium while avoiding the strong signal of the pericardium. Common tracking problems should be rectified by manual adjustment if required. LA strain is measured as GLS of the entire wall, and segmental strain is not considered. The zero reference is end-diastole, corresponding to mitral valve closure; following recommendations, we defined it by R wave at ECG (or simply the nadir of the atrial strain curve).

**Figure 1:**
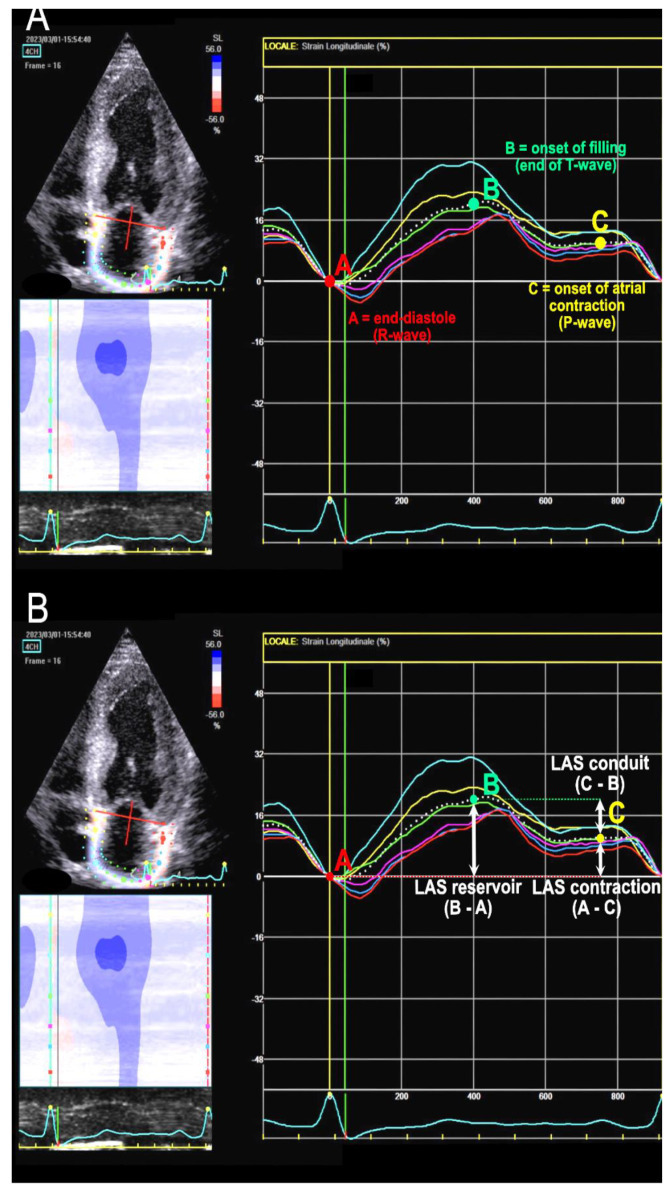
Left atrial strain (LAS) assessment. The white dotted line represents the average of the curves of the six atrial segments. (**A**): reference points for atrial function phases in relation to ECG waves and cardiac cycle. (**B**): here it is shown how to calculate the three LAS values from the reference points.

Phasic strain calculation involves calculating the deformation of atrial wall during three phases: reservoir strain (LASr), conduit strain (LAScd), and contraction strain (LASc). LASr, always positive, is calculated as the difference between the strain value at the curve peak and the end-diastolic value. LAScd, always negative, is calculated as the difference between the strain value at the onset of atrial contraction (*p*-wave) and the peak value (in atrial fibrillation (AF) patients, LAScd has the same value as LASr but with a negative sign) [15]. LASc is calculated from the difference between the strain value at end-diastole (R-wave) and the value at the onset of *p*-wave. It always exhibits a negative value and only occurs in sinus rhythm. See Figure 1 for details.

### 2.4. Clinical Outcomes Assessment

Arrhythmic episodes were reported during the patient’s medical history interview: they were documented and recorded based on the medical reports provided by the patient. Arrhythmias were detected through Holter ECG monitoring or device interrogation in patients with an implanted cardioverter-defibrillator (ICD). We included episodes of ventricular fibrillation, sustained and non-sustained ventricular tachycardia, and AF.

Exertional dyspnea was assessed during patients’ medical history interview: we inquired about the patient’s current level of exercise tolerance (ordinary and extra-ordinary physical activity) and how it has changed over the past six months, asking specifically to quantify the impact of exertional dyspnea on their daily life based on effort intensity and whether there have been any alterations in the last six months compared to prior periods. Therefore, we expressed the reports according to the New York Heart Association (NYHA) classification.

### 2.5. Ethical Considerations

This study was conducted in accordance with the principles stated in the Declaration of Helsinki. Written informed consent was obtained from all participants at the time the tests were performed, according to the indications of the hospital.

### 2.6. Statistical Analysis

Data are expressed as median and inter-quartile range (IQR) for continue variables, in consideration of the relatively small sample size, regardless of distribution, and as number and percentage for categorical variables. Data were compared using the Mann–Whitney U test between two groups as appropriate, ANOVA or Kruskal–Wallis tests for comparison among more than two groups, and chi-square and Fisher’s exact test for non-continuous variables: statistical significance was defined for *p* < 0.05, two-tailed test. We also applied the Pearson correlation and subsequently linear regression and binary logistic regression to examine the association between the echocardiographic parameters, and the association of selected clinical outcomes of interest with echocardiographic parameters. Analyses were performed using IBM SPSS Statistics ver.26 software.

## 3. Results

100 patients were included in this study, distributed as follows: 33 patients in the Cardiac Amyloidosis (CA) group, 34 patients in the Sarcomeric Hypertrophic Cardiomyopathy (HCM) group, and 33 patients in the control group (Co). In the CA group, the median age was 68 years, with 72.7% of the cohort being male. Most of the patients were categorized as NYHA class 2 (69.8%), while 21.2% were classified as NYHA class 1, and 9% were in NYHA class 3. Atrial fibrillation (AF) was reported for six patients (18.2%). Of these, four had permanent atrial fibrillation (which was also present during the examination, and therefore the atrial strain assessment was appropriately managed accordingly), while the other two reported episodes of paroxysmal atrial fibrillation in their medical history. Only one patient reported episodes of non-sustained ventricular tachycardia (NSVT).

For additional details regarding the general characteristics of the patients, please refer to Table 1.

From an echocardiographic point of view, they were all affected by left ventricular hypertrophy (median LVMi 150 g/sqm, IQR 123.5–188.5). The mean EF was 50%, and the median TAPSE was 18 mm [IQR 15–21]. These patients exhibited markers of altered diastolic function, with increased median LAVi (43.3 mL/sqm, IQR 37.4–53) and increased E/e’ ratio (median value 16.3). LV-GLS was markedly reduced (median −12%, IQR [−10, −14.2) with a typical apical sparing pattern. Atrial strain was compromised, with the following median values: LAS-reservoir 9%, LAS-conduit −6.7%, and LAS-contraction −3%.

When comparing the results with a group of patients with HCM, in CA patients we found a significantly lower LV-EF, significantly higher E/e’ ratio, and significantly lower LV-GLS (*p* = 0.005), with a different pattern of distribution of both hypertrophy (concentric vs. asymmetric) and strain alterations. Regarding atrial strain, we found significantly lower values for LAS-reservoir (*p* = 0.009), while LAS-conduit and LAS-contraction values were numerically lower but not significant (*p* = 0.09 and 0.14, respectively).

When comparing CA patients to the Co group, many of the examined parameters were significantly altered, both in terms of biventricular contractile function (significantly reduced LV-EF and tricuspid annular plane systolic excursion [TAPSE], both *p* < 0.001) and diastolic function (significantly increased LAVi and E/e’, *p* < 0.001), as well as LV-GLS (−12% vs. −19%, *p* < 0.001) (see Table 2 for further details).

**Figure 2:**
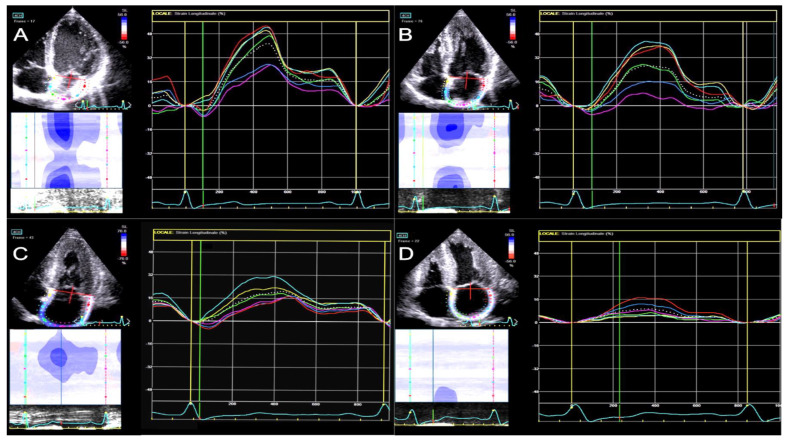
LA strain curves in comparison between subjects from the four groups. (**A**)—Co subject; (**B**)—HCM patient; (**C**)—CApEF patient; (**D**)—CArEF patient.

All atrial strain parameters were significantly impaired in CA patients than in Co (*p* < 0.001 for all three comparisons) (See Table 2 and Figure 2).

Applying Pearson’s correlation, we found a significant correlation (r > 0.5) of LAS-reservoir with LVMi, LAVi, E/e’, GLS, (negative correlation), of LAS-conduit with age and GLS (positive), and of LAS-contraction with E/e’ and GLS (positive).

Applying binary logistic regression, AF was significantly associated with LAVi (OR 1.03 95% CI 1.005–1.069, *p* = 0.025), LA-reservoir (OR 0.77 95% CI 0.641–0.925, *p* = 0.005), LA-conduit (OR 1.274, 95% CI 1.034–1.570, *p* = 0.023), and LA-contraction (OR 1.14, 95% CI 1.003–1.304, *p* = 0.046).

NSVT were associated with LAVi (OR 1.045, 95% CI 1.010–1.080, *p* = 0.010), LAS-reservoir (OR 0.77, 95% CI 0.641–0.925, *p* = 0.005), LAS-contraction (OR 1.125, 95% CI 1.006–1.259, *p* = 0.04), and LV-GLS (OR 1.193, 95% CI 1.014–1.404, *p* = 0.03).

The presence of reported exertional dyspnea was found associated with LAVi, E/e’, LAS-reservoir, LAS-conduit, LAS-contraction, TAPSE, and EF (all *p* significant).

Applying linear regression, LAS-reservoir was found associated with LV-GLS (r 0.4–0.5).

Subanalysis of patients with CA was according to ejection fraction and comparison with other groups (HCM and control group).

Subsequently, we stratified the patients with Cardiac Amyloidosis based on their ejection fraction into two groups: preserved (CApEF, N = 20) if >50% or reduced (CArEF, N = 13) if <50%. We then analyzed the differential echocardiographic characteristics between these groups. The CApEF patients were significantly younger (median age 65.5 vs. 75, *p* = 0.02) and exhibited slightly higher systolic blood pressure values compared to the CArEF. The median ejection fraction (EF) was 55.5% in the CApEF group compared to 37% in the CArEF; left ventricular global longitudinal strain (LV-GLS) was significantly lower in patients with reduced EF (*p* = 0.02). Markers of diastolic function were more impaired in the CArEF group (both E/e’ ratio and LAVi), although the difference was not statistically significant. Atrial strain parameters were also numerically reduced in the CArEF compared to the CApEF group, but except for LAS-conduit, the difference did not reach statistical significance.

**Figure 3:**
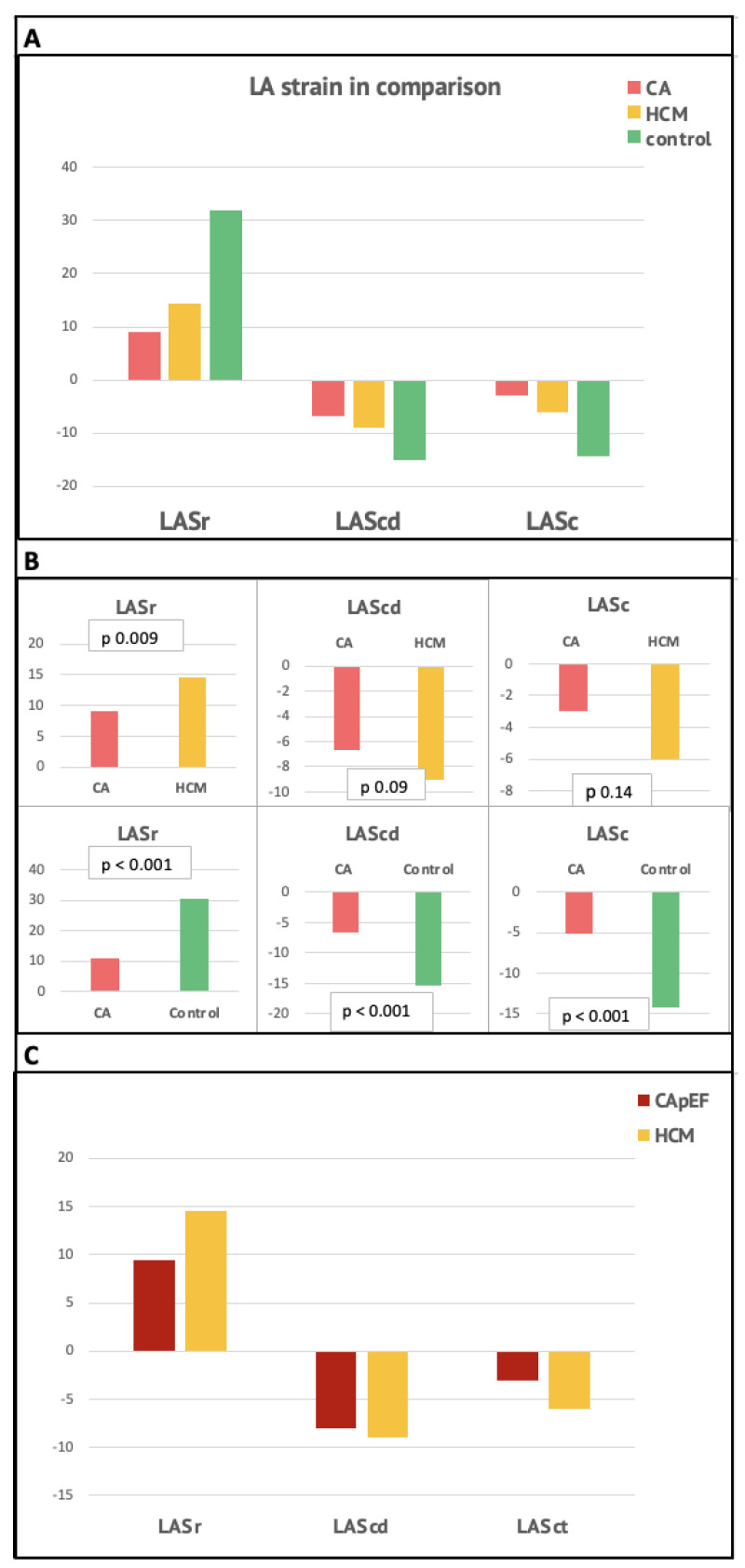
Comparison of LA strain values. (**A**)—Between the three study groups; (**B**)—between Cardiac Amyloidosis and HCM (upper panels) and between Cardiac Amyloidosis (CA) and controls (lower); (**C**)—between CApEF and HCM. Abbreviations. LASr = Left atrial strain reservoir; LAScd = Left atrial strain conduit; LASc = Left atrial strain contraction; CA = Cardiac Amyloidosis; HCM = hypertrophic cardiomyopathy; CApEF = Cardiac Amyloidosis with preserved ejection fraction.

Therefore, to differentiate and exclude patients with reduced EF, who exhibited more altered parameters potentially linked to heart failure with systolic dysfunction and its hemodynamic consequences, we decided to compare the CApEF group with the HCM group. On comparing the CApEF group with the HCM group, we found a significant difference in EF (*p* = 0.004), E/e’ ratio, s’ wave, and TAPSE. The LAS-reservoir, LAS-conduit, and LAS-contraction values were numerically lower in the CApEF group compared to the HCM group (although without statistical significance) (Table 3 and Figure 3).

## 4. Discussion

Our study assessed cardiac function and atrial strain in CA patients, comparing them to HCM patients and controls, offering valuable insights into atrial function in these populations.

CA, characterized by LV-hypertrophy, is difficult to distinguish from other LV hypertrophy-causing diseases, such as HCM and hypertensive cardiopathy. Previous studies demonstrated reductions in ventricular longitudinal strain parameters in CA patients and identified specific deformation parameters with discriminative capacity for differential diagnosis [16,17,18,19]. Our study supports these findings and expands on them by providing a detailed comparison between CA, HCM, and control subjects.

We found that CA patients had altered biventricular contractile and diastolic function, with reduced LV-GLS, increased LAVi, and elevated E/e’ ratio. Atrial strain was compromised, with lower values for LA-reservoir, LA-conduit, and LA-contraction. Sub-analysis of CA patients revealed differences in age, systolic-BP, exertion tolerance, and diastolic function markers between CApEF and CArEF groups.

Our study aimed firstly to compare HCMs patients to the whole CA patient population, at various stages of the disease. A subgroup of these CA patients had a reduced ejection fraction, indicative of a more advanced cardiac impairment with contractile dysfunction, typically seen in older patients. Our intent was to study the differing behaviors of atrial function in these two conditions, both characterized by myocardial hypertrophy and diastolic dysfunction, progressing to restrictive patterns in more advanced cases.

We subsequently isolated the subset of CApEF patients and compared them with HCMs patients, all of whom had preserved EF. This was conducted to compare two groups with similar ejection fraction, stripping away the possible repercussions on atrial dilation and dysfunction and symptoms due to compromised contractile function. The focus was to compare atrial function between these two groups, seeking differences with the aim of identifying early myocardial involvement and atrial myopathy in CA, while the ejection fraction is still preserved, to promote early initiation of therapy.

LA strain is an emerging marker of diastolic dysfunction. In Cardiac Amyloidosis, the left atrium is impaired like the left ventricle due to amyloid infiltration, leading to increased size, reduced ejection force, and strain. While LA dimension or volume can suggest chronic elevation of LA pressure, they are insufficient parameters to obtain detailed information about LA function [20].

Impaired atrial strain parameters in CA patients from our series are consistent with previous studies reporting reduced atrial function in CA, with peak LA strain correlated with LV-GLS and worse LA strain values in ATTRwt than AL [12]. A multicenter study found that LA reservoir and contraction correlated with LV-GLS and invasive LV-filling pressures, suggesting LA reservoir as an alternative marker of elevated filling pressure and LA compensation to maintain normal LV-filling pressure [21].

Aimo et al. explored multi-chamber speckle tracking imaging for assessing LA strain’s diagnostic value in CA, demonstrating altered strain parameters, particularly in ATTR-CA, and good diagnostic accuracy in differentiating CA from other unexplained LV-hypertrophies [22]. They also found an association of severe impairment of peak atrial longitudinal strain (PALS) or LASc with a diagnosis of ATTR-CA.

Another study revealed increased LA volume and reduced LA strain in ATTR-CA patients, with stronger correlation of LA strain with LA volumes, E/e’, and LV-GLS for AL-CA than ATTR-CA, possibly due to a more acute disease course and less time for amyloid deposition in the LA-wall [23]. Our study supports these findings, with lower LA strain values in CA patients, but we did not include AL-CA patients for comparison.

Studies on HCM patients identified LA strain as a significant predictor of exercise tolerance, AF risk, appropriate ICD therapy, and HF incidence [24,25,26,27]. These findings emphasize the importance of evaluating LA strain in HCM patients to improve risk stratification, early intervention, and treatment outcomes.

Significant differences in LA strain parameters between CA and HCM patients and between CA patients and controls align with previous studies aiming to differentiate these conditions based on atrial function.

Rausch et al. examined LA strain for differentiating CA from hypertensive heart disease, finding good diagnostic accuracy and a similar reduction in LA strain values between ATTR and AL groups [28].

Our study also highlighted the diagnostic potential of left atrial strain in Cardiac Amyloidosis; however, we focused mostly on the comparison with HCM, while our control, healthy, or affected by mild hypertension groups did not have significant LV hypertrophy.

It has been reported an LA strain and four-chamber (4-CH) GLS significantly reduced in CA and HCM compared with control subjects, with CA patients showing the lowest values, and LV-EF significantly reduced in CA patients in association with major adverse cardiac events (MACE), suggesting that the severity of LV systolic dysfunction could influence cardiac events and a prognostic influence of LA on MACE and AF incidence [10,29,30].

Lucas et al. demonstrated a significant difference in PALS and contraction-phase strain between the two groups CA and HCM patients [31]. LA dysfunction in CA has been shown to be likely caused by amyloid deposition in the LA wall, as confirmed by atrial wall LGE in CMR study [10,31].

Another study revealed reduced atrial deformation during atrial systole in hypertrophic ATTR-CA patients independent of LA size, unlike HCM, with LA strain rate being the only independent predictor of atrial arrhythmias [32].

### 4.1. LA Strain: Prognostic Value

Our study’s findings on LA strain parameters’ association with echocardiographic and clinical markers in CA patients align with previous research, which reported a prognostic value for LA strain and a relationship between LA strain and arrhythmia susceptibility, such as AF and NSVT.

Huntjens et al. found that peak LA strain had the strongest association with survival, and LA strain combined with LV-GLS and RV-free wall strain had the highest prognostic value in a longitudinal study of CA patients [33].

Oike et al. found reduced LA strain values in patients with cardiovascular death during follow-up and noted that LASr was independently associated with cardiovascular death and HF-related hospitalization in patients with ATTRwt [34].

In CA, LASr and LASc are often compromised, regardless of LA size [12], suggesting both raised LV-filling pressures and direct atrial amyloid infiltration contribute to dysfunction. Consequently, atrial and left appendage thrombi may develop, increasing embolic stroke risk and mortality [35,36]. The right ventricle is also commonly impacted, leading to decreased TAPSE, tissue Doppler systolic velocity, and longitudinal strain [37].

Bandera et al. found that LA infiltration was associated with greater disease severity, worse prognosis, impaired three-phasic atrial function, and “atrial electromechanical dissociation”. This phenomenon, with an absence of atrial contraction in 22.1% despite sinus rhythm, had risks and prognosis like patients with AF, worse than those with sinusal rythm and effective mechanical contraction [38]. This highlights atrial strain’s utility in detecting atrial myopathy and preventing thromboembolic complications, independently and before AF development.

### 4.2. Insights about Cut-Off Values from Literature

In the study of LA strain, various cut-off points are emerging as potential indicators of LA dysfunction. Rausch et al. proposed an LAS-reservoir cut-off value of 20%, suggesting it could aid in distinguishing Cardiac Amyloidosis from hypertensive heart disease, especially in clinically uncertain cases with increased LV wall thickness where an LAS-reservoir <20% increases the likelihood of Cardiac Amyloidosis as a differential diagnosis [28]. De Gregorio et al. offered specific cut-offs for LA reservoir and pump function (≤20.05% and ≤−1.4%, respectively) to differentiate hypertrophic phenotypes [10]. Aimo et al. suggest the first quartiles of PALS or PACS (<6.65% or <3.62%) as potential diagnostic cut-offs, particularly beneficial for patients with unexplained hypertrophy, considering the independent diagnostic value of the combination of both parameters and their ability to reclassify patient risk of ATTR-CA [22].

Regarding survival prognosis, Kado et al. pointed to a cut-off of 8.05 (peak strain) [30], while Oike indicated an optimal LAS reservoir cut-off of 6.69% for predicting cardiovascular death [34].

Cut-off values for LAS reservoir were proposed in relation to LV diastolic function, that was therefore categorized into four categories: LAS reservoir ≥35% (grade 0), ≥24% to <35% (grade 1), ≥19% to <24% (grade 2), and <19% (grade 3), with grade 2+ associated with incident heart failure in the elderly, independent of LAVI [4,39]. Inoue and Nagueh’s works emphasized an 18% cut-off for LAS reservoir to differentiate between normal and elevated LV filling pressure (when defining PCWP > 12 mmHg as elevated, and 16% when using PCWP ≥ 15 mmHg). However, they cautioned that this parameter is most accurate in estimating high filling pressure in patients with depressed EF (<50%), and less in patients with preserved EF [21,40].

At the current stage, there’s no single universal cut-off applicable to all scenarios. While useful, these cut-offs are not without limitations. The fluctuating accuracy of LAS reservoir in estimating LV filling pressures, especially in relation to ejection fraction, underscores the need for it not to be a standalone diagnostic tool. Future research should strive to refine these cut-offs, exploring their applicability across patient populations and conditions. 

### 4.3. Strengths and Clinical—Practical Implications

Our study’s notable strength is the in-depth analysis of atrial strain parameters across different CA patient subgroups based on LV-EF (preserved vs. reduced), which provides valuable insights into LV-EF’s potential influence on atrial dysfunction in CA patients, an aspect previously under-investigated. Our findings have significant clinical implications. LA strain may help clinicians differentiate CA from other conditions like HCM and hypertension; moreover, understanding LA strain parameters in CA can improve patients’ risk stratification and management and enable more personalized therapeutic approaches, such as initiating anticoagulation therapy or closer monitoring for AF development. Our study highlights the need for further research to investigate atrial strain’s prognostic implications in CA patients. Longitudinal follow-up data can provide insights into atrial strain’s role in predicting outcomes like HF, stroke, and mortality. Future research could explore treatment strategies’ impact on LA strain parameters and their potential role in monitoring treatment response. Additionally, investigating associations between LA strain parameters and echocardiographic markers, arrhythmias, and symptoms in CA patients could enhance our understanding of CA pathophysiology, contributing to novel therapeutic approaches targeting atrial dysfunction and potentially improving patient outcomes and quality of life.

### 4.4. Study Limitations and Future Perspectives

Our study has limitations, including a relatively small sample size, limiting generalizability, and a lack of longitudinal follow-up analysis to assess atrial strain parameters’ prognostic value in CA patients. Future studies with larger cohorts and longitudinal follow-up data are needed to confirm these findings and address these limitations, investigate atrial strain’s prognostic implications, and explore treatment strategies’ impact on atrial strain parameters, focusing on understanding pathophysiological mechanisms, refining diagnostic criteria, and optimizing patient management strategies.

## 5. Conclusions

In conclusion, our study contributes valuable insights into atrial function in CA patients, showing significantly LA strain parameters in ATTR-CA patients compared to HCMs and control groups (this impairment remains consistent even in the subgroup with preserved EF). These findings highlight the potential role of LA strain in differentiating between CA and other conditions, identifying patients at higher risk of arrhythmias and evaluating cardiac involvement severity and response to therapy.

## Figures and Tables

**Table 1:** General characteristic of the three groups.

	CA N = 33	HCM N = 34	Co N = 33	*p* (All Groups)	CA vs. HCM	CA vs. Co	HCM vs. Co
Age (yo)	68 (62.5–77.5)	58.5 (38.7–63.2)	58 (53–65)	<0.001	<0.001	0.001	0.37
M	24 (72.7%)	23 (67.6%)	18 (54.5%)	0.31	0.79	0.20	0.32
F	9 (27.3%)	11 (32.4%)	15 (45.5%)	0.31	0.79	0.20	0.32
NYHA 1	7 (21.2%)	18 (52.9%)		<0.001	0.01	<0.001	<0.001
NYHA 2	23 (69.8%)	14 (41.2%)		<0.001	0.03	<0.001	<0.001
NYHA 3	3 (9%)	2 (5.9%)		0.28	0.38	0.06	0.49
NYHA 4	0	0		1	1	1	1
BSA	1.8 (1.7–1.9)	1.9 (1.6–2.0)	1.9 (1.7–2)	0.48	0.24	0.30	0.69
BP-sys, mmHg	125 (112.5–135)	130 (120–140)	135 (131–145)	0.001	0.11	<0.001	0.01
BP-dia, mmHg	80 (75–85)	80 (70–85)	85 (80.5–90)	0.03	0.13	0.07	0.004
HR, bpm	71 (65–78)	65 (60–74.7)	68 (60.5–75)	0.33	0.07	0.11	0.67
Fam. history SCD	2 (6.1%)	9 (26.5%)	0	0.001	0.04	0.49	0.002
Exertional dyspnoea	29 (87.9%)	14 (41.2%)	0	<0.001	<0.001	<0.001	<0.001
Syncope	1 (3%)	2 (5.9%)	0	0.65	1	1	0.49
AF	6 (18.2%)	3 (8.8%)	0	0.03	0.30	0.02	0.24
NSVT	1 (3%)	8 (23.5%)	0	0.001	0.031	1	0.005
ICD	1 (3%)	3 (8.8%)	0	0.31	0.61	1	0.23

Values are expressed as number and percentage for categorical ones, and as mean ± SD or median and interquartile range as appropriate. Abbreviations. AF = atrial fibrillation; BSA= body surface area; BP = blood pressure; HR = heart rate; ICD = implantable cardioverter defibrillator; NSVT = non sustained ventricular tachycardia; NYHA = New York Heart Association; SCD = sudden cardiac death.

**Table 2:** Echocardiographic parameters of all groups.

	CA N = 33	HCM N = 34	Co N = 33	*p* All Groups	CA vs. HCM	CA vs. Co	HCM vs. Co
EF, %	53 (40.5–58.5)	63.5 (58–69)	60 (56.5–63)	<0.001	<0.001	<0.001	0.08
LVMi (g/sqm)	150 (123.5–188.5)	130.2 (117.2–153)	79 (68–96.5)	<0.001	0.056	<0.001	<0.001
E/e’	16.3 (11.7–21.4)	10 (7.2–14.2)	6.5 (6–8.1)	<0.001	0.001	<0.001	<0.001
LAVi (mL/sqm)	43.3 (37.4–53)	40 (31.5–57.2)	25.6 (20.6–30.5)	<0.001	0.46	<0.001	<0.001
TAPSE mm	18 (15–21)	23 (20.5–25)	23 (20–26)	<0.001	<0.001	<0.001	0.92
LV-GLS, %	−12 (−10, −14.2)	−15 (−11.7, −18)	−19 (−18, −20.5)	<0.001	0.005	<0.001	<0.001
LAS-reservoir, %	9 (5.8–16.6)	14.5 (9.7–25)	32 (25–38)	<0.001	0.009	<0.001	<0.001
LAS-conduit, %	−6.7 (−4.2, −8.6)	−9 (−4.9, −15.3)	−15 (−12.1, −18.5)	<0.001	0.09	<0.001	0.005
LAS-contract., %	−3 (−0.9, −10.5)	−6 (−3, −9.7)	−14.3 (−10.5, −19.5)	<0.001	0.14	<0.001	<0.001

Values are expressed as mean ± SD or median and interquartile range [IQR] as appropriate. Abbreviations. EF = ejection fraction; LVMi = LV mass index; E/e’ = ratio between E wave of mitral flow and e’ at Tissue Doppler; LAVi = left atrial volume index; TAPSE: tricuspid annular plane systolic excursion; LV-GLS = left ventricle global longitudinal strain; LA: left atrium.

**Table 3:** Echocardiographic parameters in the two Cardiac Amyloidosis subgroups (with preserved EF and reduced EF) and comparison of CApEF group with HCM.

	CArEF N = 13	CApEF N = 20	HCM N = 34	CApEF vs. CArEF	CApEF vs. HCM
Age, yo	75 (66.5–82)	65.5 (57–72.5)	58.5 (38.7–63.2)	0.02	0.01
BP-sys, mmHg	115 (110–131.5)	130 (123.5–135)	130 (120–140)	0.03	0.77
BP-dia, mmHg	82 (77.5–85)	80 (75–88.7)	80 (70–85)	0.78	0.28
HR, bpm	69 (63–75)	76.5 (65–78.7)	65 (60–74.7)	0.23	0.06
NYHA 1, n (%)	2 (15)	5 (25)	18 (53)	0.67	0.05
NYHA 2	9 (60)	14 (70)	14 (41)	1	0.15
NYHA 3	2 (15)	1 (5)	2 (6)	0.54	1
AF, n (%)	3 (20)	3 (15)	3 (9)	0.65	0.66
EF, %	37 (34.5–41)	55.5 (54.2–60)	63.5 (58–69)	<0.001	0.004
LVMi, g/sqm	165.5 (129.7–206.7)	147.3 (119.8–176.8)	130.2 (117.2–153)	0.25	0.24
E/e’	16.3 (13.5–22.2)	15.9 (11.4–19.6)	10 (7.2–14.2)	0.43	0.001
S’, cm/s	4 (4–5.7)	5 (5,6)	7 (5–8)	0.06	0.002
LAVi, mL/sqm	47.4 (40.1–60.5)	41.9 (28.5–50.5)	40 (31.5–57.2)	0.08	0.85
TAPSE, mm	19 (11.5–24)	17.5 (15.7–19.2)	23 (20.5–25)	0.82	<0.001
LV-GLS, %	−10 (−7, −12)	−13 (−10, −15)	−15 (−11.7, −18)	0.02	0.07
LAS-reservoir, %	6.4 (3.9−13.5)	9.4 (7–18)	14.5 (9.7–25)	0.10	0.09
LAS-conduit, %	−5.2 (−3.3, −7.2)	−8 (−5, −9.7)	−9 (−4.9, −15.3)	0.04	0.35
LAS-contract. %	−3.5 (0.4, −10.2)	−3 (−1, −11)	−6 (−3, −9.7)	0.80	0.22

Values are expressed as mean ± SD or median and interquartile range (IQR) as appropriate. Abbreviations. AF = atrial fibrillation; CArEF = Cardiac Amyloidosis with reduced ejection fraction; CApEF = Cardiac Amyloidosis with preserved ejection fraction; BP-sys = blood pressure systolic; BP-dia = blood pressure diastolic; HR = heart rate; EF = ejection fraction; LVMi = LV mass index; E/e’ = ratio between E wave of mitral flow and e’ at Tissue Doppler; LAVi = left atrial volume index; NYHA = New-York Heart Association; TAPSE = tricuspid annular plane systolic excursion; LV-GLS= left ventricle global longitudinal strain; LA = left atrium.

## Data Availability

The data presented in this study are available on request from the corresponding author. The data underlying this article will be shared on reasonable request to the corresponding author.
